# Role of *Bacillus* spp. Plant Growth Promoting Properties in Mitigating Biotic and Abiotic Stresses in Lowland Rice (*Oryza sativa* L.)

**DOI:** 10.3390/microorganisms11092327

**Published:** 2023-09-15

**Authors:** Tanja Weinand, Abbas El-Hasan, Folkard Asch

**Affiliations:** 1 Institute of Agricultural Sciences in the Tropics and Subtropics (Hans-Ruthenberg-Institute), University of Hohenheim, Garbenstr. 13, D-70599 Stuttgart, Germany; tanja.weinand@uni-hohenheim.de; 2 Department of Phytopathology, Institute of Phytomedicine, University of Hohenheim, Otto-Sander-Str. 5, D-70599 Stuttgart, Germany; aelhasan@uni-hohenheim.de

**Keywords:** PGPR, iron toxicity, brown spot disease, *Bipolaris oryzae*, abiotic and biotic stress, ACC deaminase, auxin, nutrient solubilization

## Abstract

The ability of microorganisms to promote plant growth and mitigate abiotic and biotic stresses makes them an interesting tool for sustainable agriculture. Numerous studies aim to identify new, promising bacteria isolates. Traditional culture-based methods, which focus on selecting microorganisms with plant-growth-promoting traits, such as hormone production, nutrient solubilization, and antifungal properties, are widely used. This study aims to investigate the role of plant-growth-promoting properties in bacteria-mediated stress mitigation and the suitability of traditional culture-based methods as a screening tool for the identification of beneficial bacteria. To this end, we tested three endophytic *Bacillus* isolates, which have previously been shown to affect tolerance against iron toxicity in lowland rice, (a) for their effect on the resistance against brown spot disease, and (b) for plant-growth-promoting traits using common culture-based methods. Both *B. pumilus* isolates inhibited fungal growth in vitro and reduced brown spot disease in two of three rice cultivars in planta, although they tested negative for all plant-growth-promoting traits. While *B. megaterium* was negative for ACC deaminase activity and nutrient solubilization, it exhibited auxin production. Nevertheless, *B. megaterium* did not suppress brown spot disease in any of the three rice cultivars. This study shows that bacteria do not necessarily have to possess classical plant-growth-promoting properties in order to be beneficial to plants, and it emphasizes the limitation of common culture-based methods in effectively identifying beneficial bacteria. Moreover, our results highlight the significance of the interaction between bacteria and plant cultivars in determining the beneficial effects of *Bacillus* spp. on plants under biotic or abiotic stresses.

## 1. Introduction

The use of beneficial microorganisms in sustainable agriculture has become increasingly popular. Endo- and ectophytic bacteria have been frequently shown to promote plant growth and/or increase plant tolerance against abiotic or biotic stressors [1]. Positive effects of bacterial inoculants on plant stress responses can be either direct—e.g., through bacterial phytohormone production or solubilization of nutrients—or indirect—e.g., by activation of plant transcription factors through bacterial metabolites [1,2]. Over the last decades, a broad range of bacterial inoculants have been described for their beneficial effects in numerous plant species [3]. The most common approach for screening beneficial bacteria relies on culture-based techniques. Bacteria isolated from different sources (e.g., soil or seed coats) are cultivated on various media to identify those with plant-growth-promoting traits. Bacteria showing these effects are generally known as plant-growth-promoting rhizobacteria (PGPR). Screening for bacteria strains that improve tolerance against abiotic stresses and resistance against biotic stressors often focuses on such PGPR. Plant-growth-promoting traits include the ability to solubilize tricalcium phosphates and zinc, 1-aminocyclopropane-1-carboxylic acid (ACC) deaminase activity, or siderophore and auxin production [2,4]. Through microbial solubilization of phosphates and zinc, these nutrients also become available to the plant, and hence, plant growth can be increased [2]. Plant growth reduction as a response to abiotic stress is mediated through the plant hormone ethylene [2]. However, bacterial ACC deaminase can counteract this effect by utilizing ACC, a precursor in the ethylene synthesis pathway, as substrate. This enzymatic activity decreases plant ethylene levels and subsequently alleviates growth inhibition [4]. Moreover, siderophores released by bacteria can enhance iron uptake by plants, and auxin produced by bacteria can alter the expression of genes involved in hormone production, plant defense pathways [5], and root growth [6]. Bacteria isolates lacking these essential traits are typically excluded from further evaluation.

Various biotic and abiotic stresses constrain lowland rice production worldwide. In 1942/1943, an epidemic outbreak of brown spot disease in the area of Bengal in West India caused yield losses of up to 90%. This led to the devastating Bengal famine [7]. Brown spot disease is caused by the fungus *Bipolaris oryzae* (telemorph: *Cochliobolus miyabeanus*). To this day, the fungal pathogen poses a threat to rice production. A recent study found symptoms of brown spot disease in almost 80% of the 179 rice fields investigated in western Burkina Faso [8]. One of the major abiotic stresses of lowland rice is iron toxicity. Excess uptake of iron caused by high concentrations of reduced iron (Fe II) in the soil damages the plant tissue, which, in turn, can result in complete yield losses [9]. Symptoms of brown spot disease and iron toxicity are similar to each other. Necrotic lesions develop on the leaf blades and leaf sheaths, reducing photosynthesis rates. In severe cases, entire leaves and, eventually, entire plants die. *Bacillus* spp. (consisting of two isolates of *Bacillus pumilus* and one isolate of *Bacillus megaterium*) used in this study were originally isolated from roots of lowland rice. These specific isolates were selected for their ability to reduce susceptibility to root knot nematode [10]. In a previous study, we demonstrated that inoculation with these isolates also affects the tolerance of various lowland rice cultivars to iron toxicity [11]. The objectives of the present study are (1) to investigate whether the effects of bacterial inoculants on different rice cultivars under abiotic and biotic stresses are likely to be caused by traits commonly associated with plant-growth-promoting processes, such as phytohormone production and solubilization of nutrients, and (2) to investigate if traditional culture-based methods are a suitable screening tool for the identification of bacteria isolates with the potential to mediate stress tolerance in plants. To this end, the effects of three Bacillus isolates on the sensitivity of the different rice cultivars to brown spot disease were evaluated in planta by leaf symptom scoring, as well as in vitro by testing for direct inhibitory effects on fungal growth and HCN production. Furthermore, culture-based methods were applied to test for plant-growth-promoting properties such as the production of indole compounds (IC), ACC deaminase activity, siderophore production, and the ability to solubilize zinc and tricalcium phosphates.

## 2. Materials and Methods

### 2.1. Microorganisms

Three *Bacillus* isolates (*Bacillus pumilus* D7.4; *B. pumilus* Ni9MO12 (rif.res.), hereinafter *B. pumilus* Ni9MO12; and *B. megaterium*) isolated from roots of rice seedlings [10,12] were obtained as a frozen stock in 30% (*v*/*v*) glycerol from the Institute of Crop Sciences and Resource Conservation, Department of Plant Health, Nematology and Soil Ecosystems Laboratory, University of Bonn. *Pseudomonas protegens* type strain CHAO (DSM 19095) and *Pseudomonas brassicacearum* 3Re2-7 (DSM 32001) served as reference strains in in vitro assays. *P. protegens* CHAO was used as a positive control due to its known inhibitory effects on mycelial growth on *Fusarium* sp., its siderophore production, and its nutrient solubilization ability. *P. brassicacearum* was used due to its ACC deaminase activity. The *Bipolaris oryzae* strain was kindly provided by BASF, Germany, and was multiplied on PDA. *Fusarium oxysporum* f.sp. *strigea* (FOS) strain FK3 was obtained as a frozen stock in 30% (*v/v*) glycerol from the Department of Agricultural Sciences, University of Hohenheim; *Fusarium oxysporum* f.sp. *cubense Tropical Race 4* (FOC TR4) VCG 01213/16 was obtained as a frozen stock in 30% (*v*/*v*) glycerol from the Department of Plant Pathology, Stellenbosch University, South Africa. FOS, a mycoherbicide against witchweeds (*Striga hermonthica*); FOC TR4, the causative agent of Fusarium wilt in banana; and *Bipolaris oryzae* were cultured on potato dextrose agar (PDA, Roth, Karlsruhe, Germany).

### 2.2. Plant Growth Conditions

Three rice varieties, namely, IR31785-58-1-2-3-3 (hereinafter IR31785), Suakoko 8, and Sahel 108 were selected for this study because of their known interaction with the three *Bacillus* isolates under iron toxicity [11]. Rice seeds were germinated and grown in a high-humidity plastic box on filter paper soaked in _d_H_2_O. When the 3rd leaf emerged, seedlings were transferred to a hydroponic system consisting of 7 L containers (Eurobox, Auer, Germany, 600 mm × 400 mm × 15 mm) fitted with a rack of 60 PVC tubes of 4 cm diameter and 12 cm length. Plants were grown for 21 d under greenhouse conditions with a 12 h light/dark period, mean temperatures of 25/30 °C (night/day), 50–60% relative air humidity, and a light intensity of 450–550 µmol m^−2^ s^−1^ before being subjected to further treatments. Yoshida nutrient solution [13] was renewed at weekly intervals with pH adjusted to 5.5. To adapt the plants to the nutrient solution, 25% strength was used during the first week, 50% strength during the second week, and full-strength nutrient solution until the end of the experiment.

### 2.3. Bacillus Inoculation

*B. pumilus* D7.4, *B. megaterium*, and *B. pumilus* Ni9MO12 were grown on tryptic soy agar (TSA) for 24 h at 28 °C in the dark. Single colonies were picked and propagated in liquid tryptic soy broth (TSB) at 28 °C on a shaker at 125 rpm. After overnight incubation, bacterial cells were harvested by centrifugation at 3000× *g* for 20 min, washed once with 1/4 strength Ringer’s solution, and resuspended in 1/4 strength Ringer’s solution to a final concentration of approx. 7 × 10^7^ CFU mL^−1^. Twenty-eight days after sowing, the bacteria solutions were added to the plant nutrient solution to a final concentration of approx. 10^6^ CFU mL^−1^. Control plants (non-inoculated) received the same volume of 1/4 strength Ringer’s solution. The experiment was laid out in a randomized complete block design with 6 replicates.

### 2.4. Bipolaris Oryzae Inoculation

At 35 days after sowing, plants were inoculated with *Bipolaris oryzae*. To prepare the inoculum, PDA colonized with *Bipolaris oryzae* were rinsed twice with 20 mL of sterile dH_2_O, and the mycelium, including conidia, was scraped off completely. The resulting biomass suspension was then filtered through two layers of sterile gauze. Conidia concentration was microscopically determined using a Fuchs–Rosenthal counting chamber and adjusted at 5.1 × 10^4^ conidia mL^−1^. Rice plants were sprayed with 40 mL of the conidia suspension per box using a chromatography nebulizer. Control plants were sprayed with an equal volume of dH_2_O. Subsequently, the inoculated plants were placed on wet fleece inside of a previously moistened humidity chamber.

### 2.5. Scoring of Disease Symptoms

Leaf symptoms were visually assessed 7 days after pathogen inoculation on fully expanded leaves for the entire plant based on the Standard Evaluation System for Rice (SES) (Table 1) [14].

### 2.6. Plant Harvest and Biomass Determination

For determining the dry weight, three plants from each treatment were randomly selected, separated into roots and shoots, and dried for 2 d at 70 °C in a drying chamber (ULM500, Memmert, Schwabach, Germany).

### 2.7. Detection of Siderophores

For testing the ability of *Bacillus* isolates to produce siderophores, CAS blue agar assay was used. CAS agar was prepared as described in detail in Glick (2014) [15]. Because *Bacillus* spp. are Gram-positive, they were first grown overnight on TSA plates supplemented with 0, 5, 10, and 50 ppm Fe as described above and subsequently topped with 12 mL of CAS blue agar as described by Pérez-Miranda et al. [16]. The cultures were then inoculated at 25 ± 1 °C for 21 d. Siderophore production became visible by formation of orange halos around bacterial colonies.

### 2.8. Measurement of Indole Compounds

For measurement of indole compounds produced, the method described by Sarwar et al. [17] was used. Bacteria isolates were grown in a general-purpose medium (GPM) containing 1.5 g L^−1^ glucose, 0.5 g L^−1^ ammonium sulphate, 0.5 g L^−1^ potassium hydrogen phosphate, 0.5 g L^−1^ peptone from meat, and 0.1 g L^−1^ magnesium sulphate heptahydrate at a pH 7.3, with supplementation of 0.1% L-tryptophan and different concentrations of FeSO_4_ (0, 2.5, and 5.0 ppm). Incubation was carried out at 28 °C on a shaker at 150 rpm for 4 d. Cultures were then centrifuged for 30 min at 3000× *g*. Supernatants were subsequently sterilized by passing through 0.2 µm syringe filters. Salkowsky reagent was prepared by mixing 750 µL of 0.5 M FeCl_3_ with 25 mL dH_2_O and carefully adding 15 mL of 90% H_2_SO_4_. Equal volumes of sample and Salkowsky reagent (10 mM ferric chloride in 35% chloric acid) were mixed and incubated for 30 min in the dark. A standard curve of IAA was generated using serial twofold dilutions (100, 50, 25, 12.5, 6.25, and 3.125 µM) of an IAA standard in GPM. GPM without IAA served as a blank. A separate standard curve was generated for each Fe supplementation, and the resulting equations (0 ppm Fe: y = 0.0054x + 0.1338; 2.5 ppm Fe: y = 0.0058x + 0.135; 5 ppm Fe: y = 0.0056x + 0.1454, respectively) were used for the calculation of indole compounds in the samples. Absorbance was measured at 530 nm with an Infinite 200 PRO plate reader (Infinite© 200pro, Tecan Trading AG, Männedorf, Switzerland). Aliquots of 100 µL per overnight culture were plated on TSA plates in triplicate and incubated for 12 h at 28 °C. Bacterial colonies were then counted to calculate IAA production per colony forming unit (CFU).

### 2.9. ACC Deaminase Activity

Bacterial isolates were tested for their ACC deaminase activity as described in Nascimento et al. [18] with minor modifications. *Bacillus* isolates and *Pseudomonas protegens* were grown in Minimal DF (Dworkin and Foster) salts medium [19] containing 4.0 g L^−1^ KH_2_PO_4_, 6.0 g L^−1^ Na_2_HPO_4_, 0.2 g L^−1^, MgSO_4_ × 7H_2_O, 2.0 g L^−1^ glucose, 2.0 g L^−1^ gluconic acid, and 2.0 g L^−1^ citric acid with the following trace elements: 1 mg L^−1^ FeSO_4_ × 7H_2_O, 10 mg L^−1^ H_3_BO_3_, 11.19 mg L^−1^ MnSO_4_ × H_2_O, 124.6 mg L^−1^, ZnSO_4_ × 7H_2_O, 78.22 mg L^−1^ CuSO_4_ × 5H_2_O, and 10 mg L^−1^ MoO_3_; adjusted to pH 7.2. Each isolate was grown in Minimal DF salts medium supplemented with one of the following: (a) no source of nitrogen (negative control medium), (b) 2.0 g NH_4_Cl as nitrogen source (positive control medium), or (c) 3.0 mM ACC as sole source of nitrogen (ACC test medium). Isolates which showed no growth in the negative control medium but growth in both the positive control medium and the ACC test medium were considered positive for ACC deaminase activity.

### 2.10. HCN Production Assay

HCN production was determined using a modified version of the procedure originally described by Lorck [20]. An amount of 5 mL of liquid TSB medium supplemented with 4.4 g L^−1^ of glycine for the stimulation of HCN production was inoculated with 100 µL fresh overnight bacterial culture in TSB. A sterile strip of Whatman filter paper (approx. 1 cm × 5 cm) was soaked in 0.5% picric acid in 2% sodium carbonate and then placed inside each of the culture flasks (not reaching the liquid) and fixed with the flask’s lid. The flasks were closed with parafilm and incubated for 10 days at 30 °C shaking at 150 rpm. HCN production was detected via a change in color of the filter paper strip from yellow to brownish.

### 2.11. Zinc Solubilization Assay

Bacterial isolates were tested for their ability to solubilize zinc. To that end, they were grown on Tris-mineral agar medium [21] containing 10.0 L^−1^ g D-glucose, 1 g L^−1^ (NH_4_)SO_4_, 0.2 g KCl, 0.1 g L^−1^ K_2_HPO_4_, 0.2 g L^−1^ MgSO_4_, and 15 g L^−1^ agar. The medium was supplemented with either 1.244 g L^−1^ ZnO or 1.728 g L^−1^ ZnCO_3_. An amount of 1 µL of overnight culture (in TSB) was spotted on the plates and incubated at 28 °C for 10 d in the dark. A clear halo zone was formed around colonies of zinc-solubilizing bacteria.

### 2.12. Phosphate Solubilization Assay

Pikovskaya’s Agar [22] was used for testing the ability of bacteria to solubilize phosphate. The medium contained 10 g L^−1^ D-glucose, 5 g L^−1^ Ca_3_(PO_4_)_2_, 0.5 g L^−1^ (NH_4_)SO_4_, 0.5 g L^−1^ NaCl, 0.1 g MgSO_4_ × 7H_2_O, 0.5 g L^−1^ yeast extract, 0.002 g L^−1^ MnSO_4_ × H_2_O, 0.002 g L^−1^ FeSO_4_ × 7H_2_O, and 15 g L^−1^ agar. An amount of 1 µL of overnight culture (in TSB) was spotted on the plates and inoculated at 28 °C for 10 d in the dark. A clear halo zone was formed around colonies of phosphate-solubilizing bacteria.

### 2.13. In Vitro Antifungal Activity

For antifungal activity assays, nutrient agar medium (1 g L^−1^ peptone, 1 g L^−1^ beef extract, 0.5 g L^−1^ NaCl, and 15 g L^−1^ agar) was used. A 5 mm diameter plug of actively growing fungal pathogen was placed in the center of each petri dish. An amount of 5 µL of fresh bacteria overnight cultures (in TSB) was spotted 3 cm away from the fungal disc on two opposite sides. Controls were inoculated with TSB without bacteria. Plates were incubated at 28 °C for 5–10 days in the dark. Growth of the fungus was observed daily. The diameter of the fungal growth between the bacteria colonies was measured, and the percentage of growth inhibition relative to control plates was calculated as follows:

$$\mathrm{Inhibition}\;\mathrm{rate}\;\left(\%\right)=100\;\frac{\left(C-B\right)}{C}$$

where *B* and *C* are the fungal colony diameter in the presence and absence of the bacteria, respectively [23].

### 2.14. Data Analysis

For analyzing the effect of bacterial inoculants on leaf spot development among cultivars, two-factorial ANOVA with a post hoc Dunnett’s test was carried out. To test the effect of bacteria inoculation within each cultivar, single-factor ANOVAs with post hoc Dunnett’s tests were performed. ANOVAs and post hocs were carried out in R studio version 4.0.3 “Bunny-Wunnies Freak Out” (R Foundation for Statistical Computing, Vienna, Austria). Normal distribution of data was tested by checking skewness and kurtosis in Microsoft Excel. SigmaPlot 14.0 (Systat Software Inc., San Jose, CA, USA) was used for graphs.

## 3. Results

### 3.1. Effect of Bacillus spp. on Brown Spot Disease

**Figure 1:**
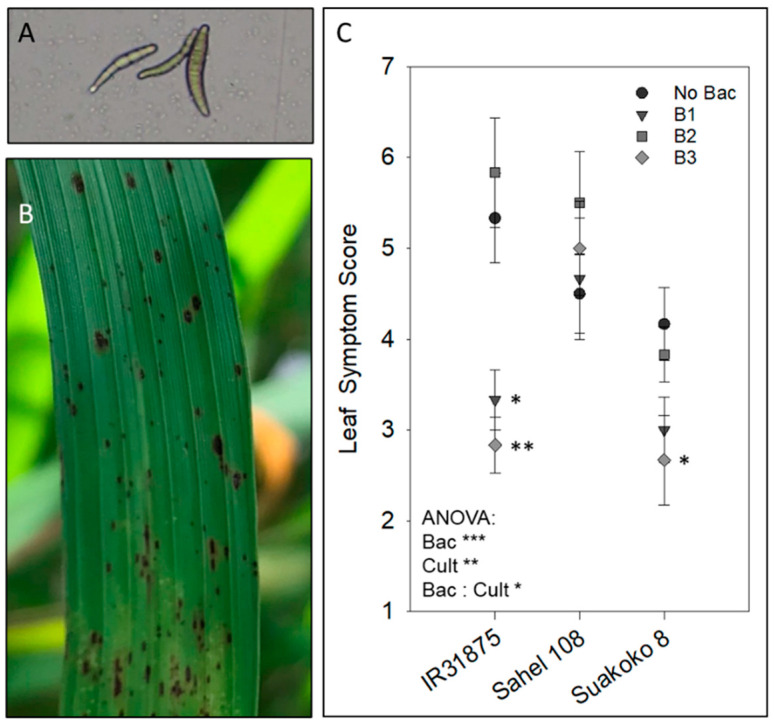
Effect of bacterial inoculation on brown spot disease. (**A**) *Bipolaris oryzae* conidia used for the infection of rice plants. (**B**) Rice leaf showing typical symptoms of brown spot disease. (**C**) The effect of bacterial inoculation on the brown leaf spot disease on three different rice cultivars. Asterisks near data points indicate significant differences compared to the noninoculated control (Dunnett’s test, α = 0.05). ANOVA = two-factorial ANOVA with bacteria (Bac) and cultivar (Cult) as factors. No Bac = no bacteria treatment, B1 = *B. pumilus* D7.4, B2 = *B. megaterium*, B3 = *B. pumilus* Ni9MO12 rif. Res.

Inoculation with *Bacillus* spp. Showed a highly significant effect on leaf symptom score among cultivars. The effects differed significantly between cultivars, and a significant interaction between bacterial isolates and rice genotype was found (Figure 1). When comparing the bacteria-inoculated plants of each cultivar to the noninoculated controls of the same cultivar, *B. pumilus* D7.4 and *B. pumilus* Ni9MO12 significantly suppressed brown leaf spot disease in IR31875 and Suakoko 8 but not in Sahel 108. However, *B. megaterium* inoculation did not reduce disease symptoms on *Bipolaris oryzae*-infected plants of all three cultivars tested.

### 3.2. HCN Production

The results obtained from HCN assay revealed that filter paper strips obtained from *Pseudomonas* culture tubes resulted in changing the color from yellow to brownish, a clear indication for HCN production. In contrast, filter paper strips incubated in the culture flasks of the three *Bacillus* isolates did not show any change in color after 10 days of incubation, and hence, they did not produce HCN (Figure S1).

### 3.3. Antifungal Activity

**Figure 2:**
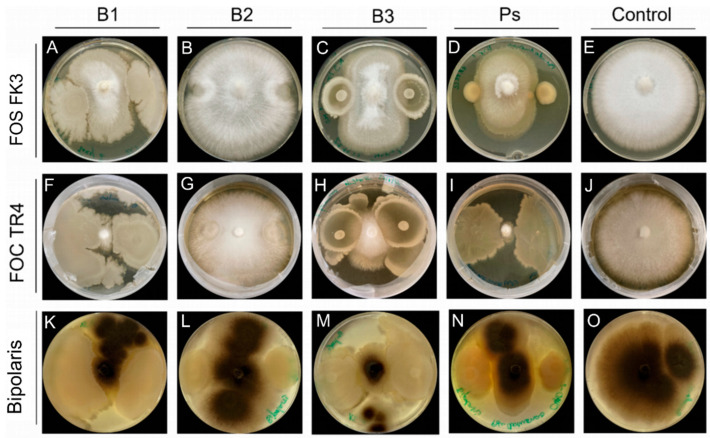
Antagonistic effects of *Bacillus* spp. on mycelial growth of *F. oxysporum* and *Bipolaris oryzae*. Bacterial cell suspension in sterile dH2O was spotted 3 cm apart on either side of the fungal agar plug, which had been placed in the middle of the plate. (**A**–**E**): FOS FK3 co-cultured with B1, B2, B3, Ps, control, respectively. (**F**–**J**): FOC TR4 co-cultured with B1, B2, B3, Ps, control, respectively. (**K**–**O**): Bipolaris co-cultured with B1, B2, B3, Ps, control, respectively. B1 = *B. pumilus* D7.4, B2 = *B. megaterium*, B3 = *B. pumilus* Ni9MO12, Ps = *Pseudomonas protegens* CHAO, Control = dH_2_O, FOS FK3 = *Fusarium oxysporum* f.sp. *strigea* strain FK3, FOC TR4 = *Fusarium oxysporum* f.sp. *cubense* Tropical Race 4, Bipolaris = *Bipolaris oryzae*.

Direct inhibitory effects of the three *Bacillus* isolates on fungal growth of different species were tested in vitro. In the presence of CHAO, mycelial growth of *F. oxysporum* FOS and FOC was inhibited by 56% and 100%, respectively (Figure 2D,I). The same bacterial strain suppressed the mycelial growth of *Bipolaris oryzae* by 60% (Figure 2N). Coculturing of *B. pumilus* D7.4 on the agar plates inhibited the mycelial growth of FOS, FOC, and *Bipolaris oryzae* by 60%, 88%, and 72%, respectively (Figure 2A,F,K). Similarly, the presence of *B. pumilus* Ni9MO12 resulted in 60%, 80%, and 72% mycelial growth inhibition of FOS, FOC, and *Bipolaris oryzae*, respectively (Figure 2C,H,M). However, cocultivation of agar plates with *B. megaterium* did not significantly affect the mycelial growth of both *Fusarium oxysporum* formae speciales tested (Figure 2B,G). Nevertheless, mycelial growth of *Bipolaris oryzae* was retarded by 44% in the presence of *B. megaterium* (Figure 2L).

### 3.4. Effects of Bacillus spp. on Plant Growth

**Figure 3:**
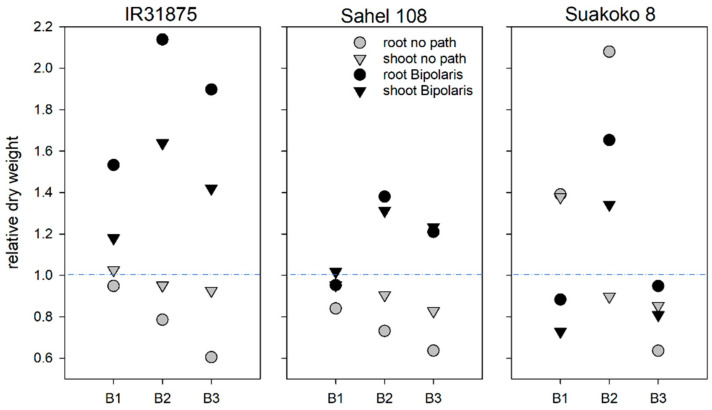
Effect of *Bacillus* inoculation on dry weight of roots and shoots of *Bipolaris oryzae*-infected and noninfected plants. B1 = *B. pumilus* D7.4, B2 = *B. megaterium*, B3 = *B. pumilus* Ni9MO12, no path = no pathogen.

Plant growth was evaluated using biomass determination of both shoots and roots. In the cases of IR31875 and Sahel 108, shoot biomass of plants not infected with the pathogen did not differ between bacteria-inoculated and noninoculated plants (Figure 3). Root biomass in noninfected and *B. pumilus* D7.4-inoculated IR31875 was also not affected, whereas in *B. pumilus*, D7.4-inoculated, noninfected Sahel 108 root biomass was reduced by 15% compared to the noninoculated, noninfected controls (Figure 3). Pathogen-infected plants of IR31875 and Sahel 108 accumulated approx. 20% less root biomass when inoculated with *B. megaterium* and about 40% less when inoculated with *B. pumilus* Ni9MO12 compared to the noninoculated, pathogen-infected controls. Non-pathogen-infected Suakoko 8 inoculated with *B. pumilus* D7.4 accumulated approx. 40% more root and shoot biomass compared to the noninoculated control. *B. megaterium* inoculation of noninfected Suakoko 8 strongly doubled root biomass but decreased shoot biomass by about 10%. Effects of *B. pumilus* Ni9MO12 inoculation on biomass of roots and shoots of Suakoko 8 were similar to those recorded in IR31875 and Sahel 108. While shoot biomass was about 10% less than in the noninoculated, noninfected control, root biomass was reduced by approximately 35%. With the exception of higher root biomass in *B. megaterium*-inoculated Suakoko 8, all differences between bacteria-inoculated, noninfected plants and non-bacteria-inoculated, noninfected plants were not statistically significant at *p* < 0.05.

In *Bipolaris oryzae*-infected IR31875, *Bacillus* inoculation in general led to a larger biomass compared to the *Bipolaris oryzae*-infected, noninoculated plants. This effect was stronger in the roots than in the respective shoots. In *Bipolaris oryzae*-infected, *B. megaterium*-inoculated plants, root biomass more than doubled and shoot biomass was 60% larger compared to the noninoculated, *Bipolaris oryzae*-infected plants.

### 3.5. Siderophore Production

**Figure 4:**
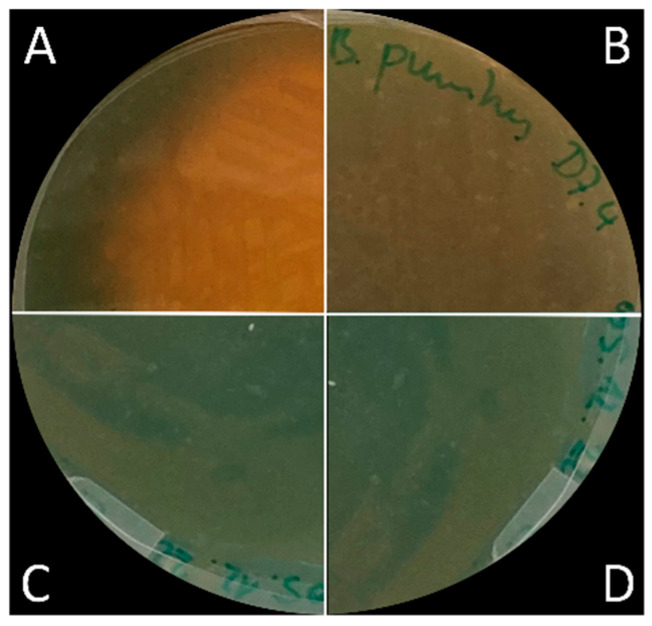
Siderophore production after 24 h. Bacteria were grown on TSA plates overnight then topped with CAS agar and incubated for 24 h. Bright orange color shows siderophore production by *Pseudomonas protegens* CHAO (**A**). Orange color development was also visible on *B. pumilus* D7.4 plates (**B**), while plates with *B. megaterium* (**C**) and *B. pumilus* Ni9MO12 (**D**) did not show any color change.

Siderophore production was monitored over three weeks. Siderophore production by *P. protegens* CHAO was so strong that the blue CAS agar started to turn orange after only 2 h and had completely turned orange after one week (Figure 4). An orange halo started forming around the colonies of *B. pumilus* D7.4 after 24 h; for *B. pumilus* Ni9MO12, the halo became visible after one week at room temperature; and for *B. megaterium*, a small halo started forming after three weeks at room temperature. When grown on TSA supplemented with different concentrations of FeSO_4_ (5 ppm, 10 ppm, and 50 ppm Fe), for all bacteria tested, a small halo around the colonies only started to develop three weeks after plates were covered with CAS agar, with no differences in halo sizes between Pseudomonas and *Bacillus* isolates on any of the iron-supplemented plates (Figure S2).

### 3.6. Auxin Production

*B. megaterium* showed the highest concentrations of indole compounds in the culture supernatants, with up to 33.8 µg mL^−1^ in the medium without iron supplementation, 11.4 µg mL^−1^ in medium supplemented with 2.5 ppm iron, and 15.8 µg mL^−1^ in medium supplemented with 5 ppm Fe (Table 2). *B. pumilus* D7.4 showed the lowest concentrations of IAA in the culture supernatants, less than 0.5 µg mL^−1^ in all three media. *B. pumilus* D7.4 showed similar concentrations of indole compounds in the supernatant as *B. megaterium*: 25.6 µg mL^−1^ in the non-iron-supplemented medium and around 11 µg mL^−1^ in the media supplemented with 2.5 ppm and 5 ppm Fe.

### 3.7. ACC Deaminase Activity

Ethylene plays an important role in the stress responses of plants. Through the activity of ACC deaminase, bacteria can alter ethylene levels and affect their tolerance against stress. Therefore, we tested the *Bacillus* isolates for their ACC deaminase activity. All three *Bacillus* isolates showed growth in the NH_4_Cl-supplemented medium, while only *P. brasscicacearum* were able to grow in medium with ACC as the only source of nitrogen. None of the three *Bacillus* showed any ACC deaminase activity (Figure S3).

### 3.8. Zinc and Phosphate Solubilization

**Figure 5:**
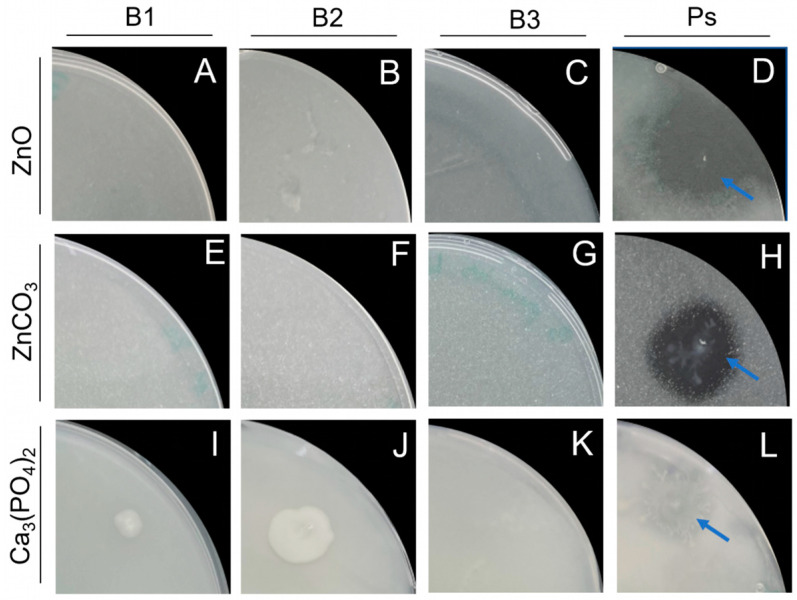
Nutrient solubilization by the different bacterial isolates. (**A**–**D**) Agar plates containing ZnO as sole Zn source; (**E**–**H**) Agar plates containing ZnCO3 as sole Zn source; (**I**–**L**) Pikowskaya agar plates containing Ca_3_(PO_4_)_2_ as sole P source. B1 = *B. pumilus* D7.4, B2 = *B. megaterium*, B3 = *B. pumilus* Ni9MO12, Ps = *Pseudomonas protegens* type strain CHAO. Blue arrows indicate halo zones around *P. protegens* colonies.

Zinc is an essential nutrient for plants, and rice grown in iron-toxic soils often shows symptoms of zinc deficiency. *P. protegens* CHAO developed clear halos around the colonies on both plates containing ZnO (Figure 5D) and ZnCO_3_ (Figure 5H) as the only source of zinc. However, neither on ZnCO_3_ nor on ZnO did *Bacillus* isolates show any Zn-solubilizing activity (Figure 5A–C,E–G).

In order to test the ability of the bacteria to solubilize Ca_3_(PO_4_)_2_, *Bacillus* isolates as well as the *Pseudomonas* control strain were grown on Pikowskayas agar plates. *B. pumilus* Ni9MO12 did not grow on Pikowskayas agar (Figure 5K). *B. pumilus* D7.4 and *B. megaterium* grew but did not show any clear halo around the colonies (Figure 5I,J). Only on plates with *P. protegens* did the medium become clearer around the colonies after 10 days of inoculation (Figure 5L).

## 4. Discussion

*Bacillus* spp. are amongst the most common bacteria studied for plant growth promotion and biocontrol properties [24]. Recently, we were able to show that *B. pumilus* D7.4, *B. megaterium*, and *B. pumilus* Ni9MO12 affect tolerance against iron toxicity in different rice cultivars [11].

In the present study, the effect of *Bacillus* spp. on the ability of different rice cultivars to withstand biotic stressors was tested in planta. Bacterial inoculation resulted in highly significant suppression of brown spot disease incited by *Bipolaris oryzae* (Figure 1). Similar to the previously described effects on the tolerance against iron toxicity [11], the effects of inoculation in any of the three isolates on the expression of brown spot disease in rice cultivars differed between the isolates and between cultivars. Significant suppression of leaf spot was observed in IR31875 and Suakoko 8 inoculated with *B. pumilus* isolates but not in Sahel 108 inoculated with any of the isolates (Figure 1). Under iron toxicity, genotypic differences in tolerance mechanisms may account for the varied effects of *Bacillus* inoculation on plant fitness across the different treatment × cultivar combinations [11]. Resistance mechanisms against fungal diseases also differ between cultivars. These differences can arise due to genetic variations and the presence of specific defense mechanisms in the respective cultivar. Specific genes may encode proteins involved in recognizing and responding to fungal invasion, such as pathogen recognition receptors (PRRs) or proteins involved in defense signaling pathways. In addition, phytohormones, such as salicylic acid, jasmonic acid, and ethylene play crucial roles in regulating plant defense responses. The balance and interaction of these hormones can differ among cultivars, influencing their resistance against specific fungal pathogens. These genotypic differences could also account for the differences seen in the effect of bacteria inoculation on brown spot disease development.

The ability to produce HCN is often included in studies screening for beneficial bacteria [25,26]. None of the isolates tested in our study showed any HCN production, and it can be concluded that the effect of bacteria inoculation on brown spot manifestation in rice is not due to the production of HCN. Nevertheless, the role of HCN in bacterially mitigated biocontrol has been questioned before, as concentrations of bacterial HCN in the rhizosphere have been shown to be too low to exhibit any detectable effect [27].

Among the three *Bacillus* isolates tested, *B. megaterium* showed the least inhibitory effect on the mycelial growth of different fungi on agar plates (Figure 2). This was consistent with in planta results. Suppression of leaf spot disease was not significant in any of the three rice cultivars when inoculated with *B. megaterium*. Both *B. pumilus* isolates showed strong inhibitory effects on fungal growth in vitro. Inoculation with these two isolates also decreased the disease severity in IR31875 and Suakoko 8. However, there was no observable effect on disease severity in Sahel 108. This leads to the hypothesis that the beneficial effects of *B. pumilus* inoculation are not solely attributed to the direct inhibition of fungal growth, but rather, other mechanisms, probably the induction of resistance, may be involved. Another plausible explanation could be that Sahel 108 is unable to interact with any of the three *Bacillus* isolates tested. Results from our previous study [11] strongly support the latter hypothesis: inoculation with *Bacillus* affected the tolerance of IR31875 and Suakoko 8 against iron toxicity, while no effects were observed in Sahel 108. To gain a deeper understanding of the underlying mechanisms governing the interaction between bacterial isolates and different rice genotypes, further research is warranted.

The potential of *Bacillus* isolates to promote plant growth was also analyzed in planta. Under regular growth conditions—without being subjected to the biotic stressor—plants did not exhibit increases in biomass either of the root or of the shoot, with the exception of Suakoko 8. In this particular cultivar, plants inoculated with *B. pumilus* D7.4 showed numerically increased growth, while those inoculated with *B. megaterium* displayed significantly larger root biomass compared to the noninoculated, noninfected control group (Figure 3). The effect of bacteria inoculation on the growth of *Bipolaris oryzae*-infected plants differed between bacteria isolates and rice cultivars. In the case of IR31875, bacteria inoculation positively affected the growth of roots and shoots. However, these effects where not significant. The effects of bacteria inoculation on *Bipolaris oryzae*-infected Sahel 108 were only small and not significant. *B. pumilus* D7.4 and *B. pumilus* Ni9MO12 inoculation of Suakoko 8 infected with *Bipolaris oryzae* resulted in numerically lower biomasses compared to the noninoculated controls. This was despite the beneficial effects on brown spot disease of the two isolates in this particular rice cultivar. Only inoculation with *B. megaterium* led to significantly larger root and shoot biomass of *Bipolaris oryzae*-infected compared to non-bacteria-inoculated *Bipolaris oryzae*-infected Suakoko 8. However, this *Bacillus* isolate did not demonstrate any significant effects on leaf spot disease development. Effects of *Bacillus* inoculation on the disease reduction on the three lowland rice cultivars does not seem to be related to biomass accumulation. This is consistent with observations we previously described for plants grown under iron toxicity. The effects of *Bacillus* inoculation on biomass accumulation of the three rice cultivars grown under iron-toxic conditions could not be correlated to the effects of *Bacillus* inoculation on leaf symptoms scores [11]. Moreover, the effects of bacterial inoculation on the growth of the three cultivars appeared to be influenced by varying environmental conditions and may differ between experiments [11].

The ability of bacteria to solubilize nutrients is considered a plant-growth-promoting trait that is routinely tested in studies aiming to identify bacteria which can neutralize certain environmental stresses [1]. Iron toxicity is a multinutrient disorder that often leads to deficiencies in phosphate and zinc [9]. We tested *Bacillus* isolates for their ability to solubilize zinc and phosphate. In the hydroponic system used for testing the effect of bacteria inoculation on the tolerance of different rice cultivars against iron toxicity and brown spot disease, plants were supplied with sufficient amounts of nutrients. However, the pH of the nutrient solution decreased due to root activity. Phosphate availability decreases at low pH and is related to the reduction of iron in the soil [28]. P deficiency in rice leads to an increased exudation of organic metabolites which, in turn, increases the availability of P through microbial reduction of iron phosphates [9]. Under iron toxicity, plaques of iron form on the root surface of lowland rice. This can impair the uptake of zinc, leading to Zn deficiency [9]. None of the three *Bacillus* isolates tested in this study showed the ability to solubilize phosphate or zinc in vitro. Whether nutrient uptake of rice plants could be improved by inoculation with any of the three *Bacillus* isolates would require further in planta investigations.

Siderophores serve as high-affinity iron chelators, binding to iron ions and forming stable complexes that can be taken up by the producing organism. Under iron-limited conditions, bacterial siderophores have been shown to improve iron acquisition in rice and other plant species [1]. The role of bacterial siderophores and their potential significance under iron toxic soil conditions remains unknown. However, it has been demonstrated that bacterial siderophores play a crucial role in disease suppression. *Pseudomonas fluorescens* mutants that lost their ability to produce siderophores also lost their potential to suppress leaf blast in rice [29]. It is believed that siderophores trigger unknown signals in the roots to be transmitted to the shoots leading to ET/JA-dependent priming of plant defenses [30]. The abilities of the three *Bacillus* isolates to produce siderophores were assessed under different iron concentrations. Out of the *Bacillus* isolates tested, only *B. pumilus* D7.4 clearly showed siderophore production under normal growth conditions (Figure 4). Siderophore production of *B. pumilus* Ni9MO12 only became visible after 3 weeks, possibly when iron in the growth medium became so limited that siderophore production was induced or because Fe^3+^ was oxidized due to other bacterial activity. Unsurprisingly, with increasing iron concentrations in the growth medium, siderophore production of all strains tested was suppressed (Figure S2). As *B. pumilus* D7.4 produced siderophores under normal growth conditions, it is possible that these siderophores play a role in the suppression of brown spot disease in rice either by triggering signals that lead to a priming of plant defenses or by depriving pathogens from iron, leading to a phenomenon described as “nutritional immunity” [31].

To our knowledge, the involvement of siderophores in suppression of brown spot disease has not been demonstrated before. Therefore, further research is needed to test this hypothesis thoroughly and establish any potential relationship between siderophores and the suppression of brown spot disease.

Based on our results, we can exclude the possibility that any of the *Bacillus* isolates used in our study affect iron acquisition of rice through siderophore production under iron-toxic conditions. Our results, however, do not rule out that other alterations in iron homeostasis could be behind the effects of *Bacillus* on the plant’s response to iron toxicity and infection with *Bipolaris oryzae*. Local accumulation of Fe within the plant can be toxic to the invading pathogen [32]. This strategy requires targeted transport of iron. Previous studies have shown iron transporters of the NRAMP and the YSL families to be involved in the immune response of rice infected with *Magnaporthe oryzae* [33]. These transporters are needed for the transport of iron within the plant and were hypothesized to be affected by *B. pumilus* inoculation in Suakoko 8 [11]. Preliminary results showed that iron might also be involved in the suppression of brown leaf spot disease by *Bacillus* isolates. Perls-DAB staining of leaf blades revealed spots of iron accumulation in plants inoculated with *B. pumilus*, while in the noninoculated plants, no such iron accumulation was observed [34]. Further investigation is needed to decipher the role of iron translocation and sequestration in the plant’s defense against both *Bipolaris oryzae* infection and iron toxicity and the possible effects of *Bacillus* inoculation on these mechanisms.

Cultures of *B. megaterium* contained the highest concentrations of indole compounds of all three *Bacillus* isolates tested. The highest concentration was released in *B. megaterium* cultures without iron supplementation. The same was true in case of *B. pumilus* Ni9MO12. Similarly, the concentration of indole compounds in the supernatants of cultures without iron supplementation was the highest. *B. pumilus* D7.4 only produced small amounts of auxin, which were at the limit of detection, despite generally showing the strongest growth in the medium used. In contrast, *B. megaterium* and *B. pumilus* Ni9MO12 showed the least growth without Fe supplementation but produced the highest amount of indole compounds. In media supplemented with iron, their auxin production decreased, whereas growth was stronger. These results suggest that bacterial isolates which grow well in the medium used for screening for auxin production are likely to produce smaller amounts of indole compounds despite their general ability to do so. *B. megaterium* showed the highest level of auxin production among the isolates tested. Additionally, this particular isolate exhibited a growth-promoting effect on both the roots and the shoots of Suakoko 8 (Figure 2) when compared to the noninoculated, iron-stressed plants. Both findings are consistent with previous research demonstrating that bacterial auxin production has been shown to promote the growth, particularly of roots, of various plant species [35]. However, among the three isolates tested here, *B. megaterium* is the one that showed the least effect on the tolerance of lowland rice cultivars against iron toxicity [11] and the least effect on resistance of lowland rice against brown spot disease. Auxin does not seem to be involved in the signaling cascades triggered by *Bacillus* inoculation.

Although the smallest of the plant hormones, ethylene has been shown to play a pivotal role as a signaling molecule in the plant’s response to both abiotic and biotic stresses [36]. Upon exposure to abiotic stress conditions or pathogen attack, plants increase the production of ethylene, which leads to a promotion of growth and elongation of the cells of young leaves. 1-aminocyclopropane-1-carboxylate (ACC) is the immediate precursor of ethylene. ACC can be hydrolyzed and degraded into α-ketobutyrate and ammonia by the enzyme ACC deaminase. Many PGPR have been shown to possess ACC deaminase activity. In this way, they can decrease the ethylene level in plants, which, in turn, mitigates growth inhibition under stress [4]. This phenomenon is widely referred to as “plant growth promotion”. For accumulative stresses, such as iron toxicity or salinity, promotion of plant growth could be useful due to dilution effects; for deficit stresses, such as drought or fungal pathogens, promotion of plant growth could have negative effects, for it would use up energy needed for stress responses. We therefore suggest that it is important to strictly discriminate between actual growth promotion and mitigation of growth inhibition.

Ethylene also plays a role in the tolerance of lowland rice against iron toxicity. It induces the production of aerenchyma, a gas-conducting tissue, through which molecular oxygen is channeled from the atmosphere through the stems into the roots and into the rhizosphere, where the oxygen leads to the oxidation of Fe^2+^ [9]. However, none of the three *Bacillus* isolates tested in this study showed any ACC deaminase activity. The effects they have on the suppression of brown leaf spot disease and tolerance against iron toxicity may not be caused by directly decreasing the ethylene level. Earlier reports have also indicated that ethylene does not appear to have any influence on the resistance or susceptibility of rice against brown spot disease. However, the defense mechanisms against this disease involve signaling through abscisic acid (ABA) [37,38]. ABA is also a key player in the response to abiotic stresses. It has been shown to be involved in the plant responses to drought, cold, salinity, and heavy metals [39]. Inoculation of rice with an ABA-producing, seed-borne endophytic *Bacillus amyloliquefaciens* isolate RWL-1 led to increased salt tolerance of the plants [40]. Further investigation into possible ABA production of the three *Bacillus* isolates described here and/or into indirect effects of inoculation on ABA signaling in the different rice cultivars under iron toxicity and after infection with *Bipolaris oryzae* is still needed.

## 5. Conclusions

When searching for beneficial bacteria from any given microbiome, methods are needed for narrowing down the number of isolates. However, our results show that beneficial bacteria cannot always be detected by traditional culture-based methods. Some beneficial isolates might not possess any of the traits selected for, while others might only display them under certain environmental conditions. The selection of beneficial microorganisms for use in sustainable agricultural practices should, therefore, be based on the comparison of natural microbiomes from tolerant and sensitive cultivars rather than on “plant growth promoting” traits alone. Furthermore, the effect of bacterial inoculation strongly depends on the rice genotypes. Efforts to develop artificial microbiomes should go hand in hand with breeding strategies that consider the genotypic ability to acquire—and sustain—beneficial microbiomes.

## Figures and Tables

**Table 1:** Leaf symptom scoring according to the Standard Evaluation System for Rice (SES).

Scale (Affected Leaf Area)	
1	No incidence
2	Less than 1%
3	1–3%
4	4–5%
5	11–15%
6	16–25%
7	26–50%
8	51–75%
9	76–100%

**Table 2:** Production of indole compounds in liquid cultures of *B. pumilus* D7.4, *B. megaterium,* and *B. pumilus* Ni9MO12 bacteria grown in a general-purpose medium (GPM) for 4 d at 28 °C and shaking at 125 rpm. The medium was supplemented with 0 ppm, 2.5 ppm, and 5.0 ppm Fe in the form of FeSO_4_. Values show the mean of three independent cultures with the standard error of the mean.

	Total Indole Compounds µg mL^−1^		
	0 ppm Fe	2.5 ppm Fe	5.0 ppm Fe
*B. pumilus* D7.4	6.43 ± 0.28	3.83 ± 0.15	7.07 ± 0.57
*B. megaterium*	33.88 ± 1.01	11.4 ± 0.55	15.82 ± 1.54
*B. pumilus* Ni9MO12	28.69 ± 0.07	10.04 ± 0.74	8.49 ± 0.81

## Data Availability

All data supporting the findings of this study are available from the corresponding author upon reasonable request.

## Supplementary Materials

The following supporting information can be downloaded at: https://www.mdpi.com/article/10.3390/microorganisms11092327/s1, Figure S1: HCN production; Figure S2: Siderophore production after 21 days; Figure S3: ACC deaminase activity.
