# Supplementary material for: Role of Bacillus spp. Plant Growth Promoting Properties in Mitigating Biotic and Abiotic Stresses in Lowland Rice (Oryza sativa L.)
Source: Microorganisms. 2023 Sep 15;11(9):2327. doi: 10.3390/microorganisms11092327 (PMC10536376; doi:10.3390/microorganisms11092327)
Supplement: Supplementary file 1 [file microorganisms-11-02327-s001.zip › microorganisms-2561138-supplementary.pdf]

microorganisms

Role of *Bacillus* spp. Plant Growth Promoting Properties in Mitigating Biotic and Abiotic Stresses in Lowland Rice (*Oryza sativa* L.)

Supplementary Material

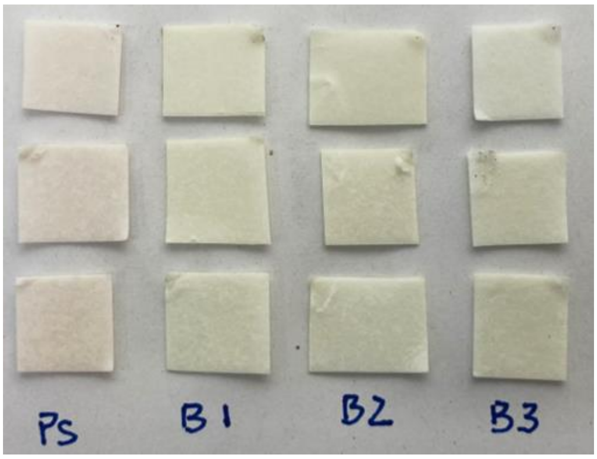

Figure S1. HCN production. Color of Whatman filter paper changed from yellow to brownish in culture flasks with *Pseudomonas protegens* growing in TSB supplemented with glycine. Filter papers stayed yellow in flasks with Bacillus cultures in TSB supplemented with glycine and in medium without bacteria inoculation. Ps = *Pseudomonas protegens* CHAO; B1 = *B. pumilus* D7.4; B2 = *B. megaterium*; B3 = *B. pumilus* Ni9MO12.

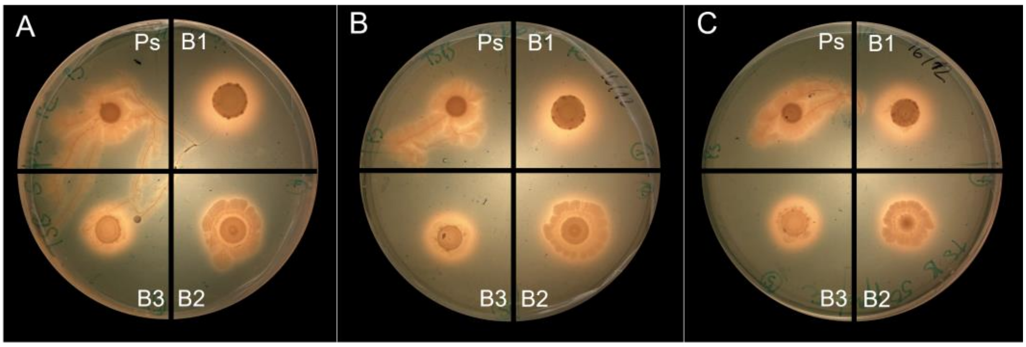

Figure S2. Siderophore production after 21 days on medium supplemented with $FeSO_4$. Orange halos developed around all colonies independent of the concentration of supplemented iron and the bacteria isolate. A = TSA supplemented with 5 ppm Fe, B = TSA supplemented with 10 ppm Fe, C = TSA supplemented with 50 ppm Fe. Ps = *Pseudomonas protegens* CHAO; B1 = *B. pumilus* D7.4; B2 = *B. megaterium*; B3 = *B. pumilus* Ni9MO12.

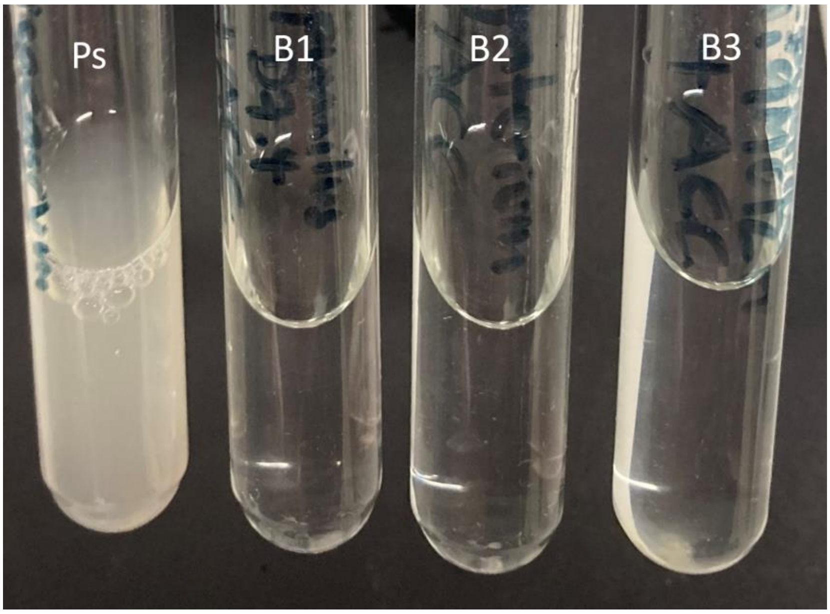

Figure S3. ACC deaminase activity. Bacteria growth in minimal DF salts medium with ACC as sole source for nitrogen. Only *Pseudomonas protegens* CHAO was able to grow in this medium. Bacillus isolates showed no growth and are therefore considered negative for ACC deaminase activity. Ps = *Pseudomonas protegens* CHAO; B1 = *B. pumilus* D7.4; B2 = *B. megaterium*; B3 = *B. pumilus* Ni9MO12.
